# Stickstoffdioxid – Bestimmung von Stickstoffdioxid in der Luft am Arbeitsplatz mittels Ionenchromatographie (IC): Luftanalysen-Methode

**DOI:** 10.34865/am1010244d10_1or

**Published:** 2025-03-31

**Authors:** Christian Monsé, George Dragan, Ulrich Prott, Christoph Emmel, Ralph Hebisch, Andrea Hartwig, MAK Commission

**Affiliations:** 1 Institut für Prävention und Arbeitsmedizin der DGUV (IPA) Bürkle de la camp-Platz 1 44789 Bochum Deutschland; 2 Bundesanstalt für Arbeitsschutz und Arbeitsmedizin (BAuA) Friedrich-Henkel-Weg 1–25 44139 Dortmund Deutschland; 3 Berufsgenossenschaft der Bauwirtschaft (BG BAU) Am Knie 6 81241 München Deutschland; 4 Institut für Angewandte Biowissenschaften. Abteilung Lebensmittelchemie und Toxikologie. Karlsruher Institut für Technologie (KIT) Adenauerring 20a, Geb. 50.41 76131 Karlsruhe Deutschland; 5 Ständige Senatskommission zur Prüfung gesundheitsschädlicher Arbeitsstoffe. Deutsche Forschungsgemeinschaft, Kennedyallee 40, 53175 Bonn, Deutschland. Weitere Informationen: Ständige Senatskommission zur Prüfung gesundheitsschädlicher Arbeitsstoffe | DFG

**Keywords:** Stickstoffdioxid, Luftanalysen, Analysenmethode, Arbeitsplatzmessung, Gefahrstoff, Ionenchromatographie, Leitfähigkeitsdetektion, IC, Aluminiumoxid, Flüssigdesorption

## Abstract

The working group “Air Analyses” of the German Senate Commission for the Investigation of Health Hazards of Chemical Compounds in the Work Area (MAK Commission) developed and verified the presented analytical method. It is used to determine the levels of nitrogen dioxide [10102-44-2] that occur in the workplace air. The method covers concentrations in the range from one tenth up to twice the current occupational exposure limit value (OELV) of 0.95 mg/m^3^ (0.5 ml/m^3^). Samples are collected by drawing a defined volume of air through a sampling tube filled with aluminium oxide carrier material coated with triethanolamine (TEA) using a flow regulated pump at a volumetric flow rate of 1.8 l/min. The exposure during the shift is assessed with a sampling period of 2 hours and the short-term exposure with a period of 15 minutes. Nitrogen dioxide reacts with TEA with formation of TEA nitrite and TEA nitrate. Nitrite and nitrate are extracted with ultra-pure water and analysed by ion chromatography using conductivity detection. The quantitative determination is based on multiple-point calibrations with external standards. A relative limit of quantification (LOQ) of 0.009 mg/m^3^ is obtained for an air sample volume of 216 litres. As the LOQ for a sample volume of 27 litres is well below 0.95 mg/m^3^, the short-term exposure limit (STEL; excursion factor 2) can also be measured. The mean recovery is 108% and the expanded uncertainty is below 28% for a sampling period of 2 hours.

**Table:** 

**Methodennummer**	1
**Anwendbarkeit**	Luftanalyse
**Analyt. Messprinzip**	Ionenchromatographie mit Leitfähigkeitsdetektion (IC)

## 1 Kenndaten des Verfahrens

**Table:** 

**Präzision:**	Standardabweichung (rel.):	*s* = 4–5 %
	Erweiterte Messunsicherheit:	*U* = 28 %
	in einem Konzentrationsbereich von 0,095–1,9 mg/m^3^ (0,05–1 ml/m^3^) und n = 6 Bestimmungen	
**Bestimmungsgrenze:**	1,9 µg absolut
	0,009 mg/m^3^ (0,005 ml/m^3^) bei einem Probeluftvolumen von 216 l und einer Probenahmedauer von 2 h	
**Wiederfindung:**	*η* = 99–115 %	
**Probenahmeempfehlung:**	Probenahmedauer:	2 h
	Probeluftvolumen:	216 l
	Volumenstrom:	1,8 l/min
	Für Kurzzeitmessungen:	15 min; 1,8 l/min

## 2 Stoffbeschreibung

### Stickstoffdioxid [10102-44-0]

**Abb.1:**
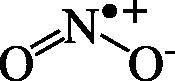
Strukturformel von Stickstoffdioxid

Stickstoffdioxid (siehe Abbildung 1, auch Stickstoff(IV)oxid oder Stickstoffperoxid genannt) ist bei 20 °C eine rotbraune, giftige, stechend chlorähnlich riechende Flüssigkeit. Aufgrund des sehr hohen Dampfdruckes von 0,96 hPa und des niedrigen Siedepunktes von nur 21,1 °C liegt es am Arbeitsplatz in aller Regel gasförmig vor. Stickstoffdioxid steht mit dem farblosen Distickstofftetroxid in einem druck- und temperaturabhängigen Gleichgewicht. Bei niedrigen Temperaturen und/oder höheren Drücken, z. B. in Druckgasflaschen, liegt in erster Linie das diamagnetische Dimer vor (IFA 2024).

Stickstoffdioxid ist nicht brennbar, ist jedoch ein starkes Oxidationsmittel und reagiert mit vielen Substanzen sehr heftig bzw. explosiv. Es wird industriell im Ostwald-Verfahren durch die katalytische Verbrennung von Ammoniak synthetisiert und für die Herstellung von Salpetersäure durch seine Hydrolyse verwendet. Bei technischen Verbrennungs­vorgängen, z. B. in Verbrennungsmotoren, insbesondere in Dieselmotoren, entsteht unbeabsichtigt neben anderen Stickoxiden hauptsächlich Stickstoffdioxid. Aber auch beim Schweißen, in Lichtbogenöfen und bei der Glasherstellung werden Stickoxide freigesetzt (IFA 2024; RÖMPP-Redaktion und Sitzmann 2010).

Der Arbeitsplatzgrenzwert (AGW) von Stickstoffdioxid beträgt 0,95 mg/m^3^ (0,5 ml/m^3^). Der Kurzzeitwert ist der Spitzenbegrenzungs-Kategorie I mit dem Überschreitungsfaktor 2 zugeordnet (AGS 2024). Der MAK-Wert ist identisch zum AGW mit einer Spitzenbegrenzungs-Kategorie I und dem Überschreitungsfaktor 1 (DFG 2024). Stoffdaten zu Stickstoffdioxid können der Tabelle 1 entnommen werden.

**Tab.1:** Stoffdaten zu Stickstoffdioxid (IFA 2024)

Name	Stickstoffdioxid
CAS-Nr.	10102-44-0
Molmasse [g/mol]	46,01
Aggregatzustand bei 20 °C	flüssig
Dichte bei 20 °C [g/cm^3^]	1,45
Dampfdruck bei 20 °C [hPa]	960
Schmelzpunkt [°C]	–11,2
Siedepunkt bei 1013 hPa [°C]	21,1
Flammpunkt [°C]	–
Beurteilungsmaßstäbe	
AGW, MAK-Wert, Deutschland (AGS 2024; DFG 2024)	0,95 mg/m^3^ (0,5 ml/m^3^)
Spitzenbegrenzungskategorie (Überschreitungsfaktor (AGS 2024) / (DFG 2024)	I(2) / I(1)

## 3 Grundlage des Verfahrens

Das Analysenverfahren ermöglicht die Bestimmung des Gehaltes an Stickstoffdioxid in der Luft am Arbeitsplatz im Bereich von einem Zehntel bis zum Doppelten des derzeit gültigen Arbeitsplatzgrenzwertes (AGW) von 0,95 mg/m^3^ (0,5 ml/m^3^) (AGS 2024). Auch die Einhaltung der Spitzenbegrenzung mit einem Überschreitungsfaktor von 1 (MAK-Wert) kann überprüft werden (DFG 2024; DIN 2021 a).

Zur Probenahme wird mit einer geeigneten Probenahmepumpe ein definiertes Luftvolumen aus dem Atembereich durch ein Probenahmesystem gesaugt. Das Probenahmesystem besteht aus einem Glasröhrchen gefüllt mit einem Al_2_O_3_-Trägermaterial, das mit Triethanolamin (TEA) imprägniert ist. Das Stickstoffdioxid reagiert mit dem TEA zu TEA-Nitrit und TEA-Nitrat und wird auf diese Weise an das Sorbens gebunden (Cape 2009). Das mit dem Stickstoffdi­oxid beaufschlagte Probenahmeröhrchen (TEA-Nitrit und TEA-Nitrat) wird in ein 50-ml-Kunststoffgefäß überführt, mit Reinstwasser überschichtet und geschüttelt. Die analytische Bestimmung erfolgt mittels Ionenchromatographie (IC) mit Leitfähigkeitdetektion. Die quantitative Auswertung erfolgt für das Nitrit- und Nitratsignal anhand von zwei getrennten Mehrpunktkalibrierungen mit externen Standards. Die Gehalte der beiden Ionen werden zur Berechnung der Luftkonzentration von Stickstoffdioxid aufsummiert.

## 4 Geräte, Chemikalien und Lösungen

### 4.1 Geräte

Für die Vorbereitung der Probenträger:

- Laborofen zum Ausglühen des Al_2_O_3_ bei 850 °C
- Tiegel oder Glühschälchen aus Porzellan, geeignet bis min. 850 °C
- Einmalspritzen aus Polyethylen (PE) mit Luer-Anschluss, 10 ml (z. B. BD Discardit II, Fa. Becton Dickinson and Company, Warwick, RI, USA)
- Rotationsverdampfer mit temperierbarem Wasserbad und einem 1-l-Rotationsverdampferkolben (z. B. Rotavapor R-210 mit B-491 Wasserbad, Fa. Büchi Labortechnik GmbH, 45127 Essen)
- Vakuumpumpe, Endvakuum min. 20 mbar (z. B. MZ 2C + AK + EK, Fa. Vakuubrand GmbH & Co. KG, 97877 Wertheim)
- Vorratsgefäße für das Al_2_O_3_-Trägermaterial aus Glas mit Schraubdeckel
- Laborwaage, 0,1–1000 g Wägebereich
- 50-ml-Becherglas und ggf. Glastrichter zum Einfüllen des Trägermaterials
- Glasröhrchen, Länge (L) 67,2 mm, Außendurchmesser (AD) 20 mm, Innendurchmesser (ID) 15 mm, Stopfen mit Bohrung aus Polytetrafluorethylen (PTFE) mit zwei kleinen passenden Viton-O-Ringen, zwei Stützsiebe aus Kunststoff mit je zwei passenden Viton-O-Ringen (z. B. Glasröhrchen: Art.-Nr. 777215-20, PTFE-Stopfen: Art.-Nr. 777217-20, kleine Viton-O-Ringe: Art.-Nr. 777218, Stützsiebe: Art.-Nr. 30003043-500, Viton-O-Ringe: Art.-Nr. 777213, Fa. LABC-Labortechnik Zillger KG, 53773 Hennef) Füllmenge: min. 4,0 g, max. 4,5 g
- Verschlusskappen aus PE (z. B. GPN 350/28 Fa. Pöppelmann GmbH & Co. KG, Bakumer Str. 73, 49393 Lohne)

Für die Probenahme:

- Probenahmepumpe für personengetragene und stationäre Probenahmen, geeignet für einen Volumenstrom von 1,8 l/min (z. B. GilAir Plus, vertrieben durch Fa. DEHA Haan & Wittmer GmbH, 71296 Heimsheim)
- Massendurchflussmesser für einen Volumenstrom von 0–20 l/min (z. B. TSI Flowmeter 4146, TSI GmbH, 52068 Aachen)
- Steifer Verbindungsschlauch z. B. aus Polyurethan mit 8 mm Außendurchmesser
- Verbindungsschlauch zur Pumpe z. B. aus Silikon mit 8 mm Innendurchmesser

Für die Probenvorbereitung und analytische Bestimmung:

- Reinstwasseranlage (z. B. Millipore-Q-Gradient mit Elix 3UV, Fa. Merck KGaA, 64293 Darmstadt)
- Messkolben, 10 ml, 200 ml und 2000 ml (z. B. Fa. Brand GmbH + Co. KG, 97877 Wertheim)
- Flaschenaufsatz-Dispenser, 10 ml oder 25 ml (z. B. Dispensette III, Fa. Brand GmbH + Co. KG, 97877 Wertheim)
- Variable Kolbenhubpipetten, 10–100 μl und 100–1000 μl (z. B. Reference 2, Fa. Eppendorf SE, 22339 Hamburg)
- Variable Kolbenhubpipette, 500–5000 µl (z. B. Fa. Brand GmbH + Co. KG, 97877 Wertheim)
- Einmalspritzen aus PE mit Luer-Anschluss, 10 ml (z. B. BD Discardit II, Fa. Becton Dickinson and Company, Warwick, RI, USA)
- Einmalaufschlussgefäß, 50 ml, mit Deckel aus PE (z. B. DigiTube, Fa. S-prep GmbH, 88662 Überlingen)
- Pinzette
- Antistatikgerät (z. B. Anti-Static Ionizer, Fa. CEM Corporation, Matthews, NC, USA)
- Einmalkanülen, 1,2 × 40 mm (z. B. BD Microlance 3, Fa. Becton Dickinson and Company, Warwick, RI, USA)
- Spritzenvorsatzfilter mit Luer-Anschluss und PTFE-Membran, Ø 25 mm, Porengröße 0,45 μm (z. B. Chromafil Xtra H-PTFE-45/25, Ref: 729246, Fa. Macherey-Nagel GmbH & Co. KG, 52355 Düren)
- Autosamplergefäße aus Polypropylen, 2,5 ml mit perforiertem Stopfen (z. B. Art-Nr. 6.2743.040 und Art-Nr. 6.2743.070, Fa. Metrohm Deutschland GmbH & Co. KG, 70794 Filderstadt)
- Ionenchromatograph mit Entgaser, Säulenofen, Autosampler, chemischer und CO_2_-Suppression und Leitfähig­keitsdetektor (z. B. 930 Compact IC Flex, Fa. Metrohm Deutschland GmbH & Co. KG, 70794 Filderstadt)
- Anionentrennsäule mit Vorsäule (z. B. Metrosep A Supp 5-250/4.0, Art-Nr. 6.1006.530 mit Metrosep A Supp 5 Guard/4.0, Art-Nr. 6.1006.500, Fa. Metrohm Deutschland GmbH & Co. KG, 70794 Filderstadt)

### 4.2 Chemikalien

- Al_2_O_3_-Trägermaterial, 1,2 mm (z. B. Art.-Nr. SA62240, Accu sphere, Fa. Saint-Gobain NorPro, Stow, OH, USA)
- Triethanolamin, p. a. 99 % (z. B. Fa Merck KGaA, 64293 Darmstadt)
- Schwefelsäure, 2,5 mol/l (5N) in wässriger Lösung, zur Suppressor-Regeneration (z. B. AVS TITRINORM, Art.-Nr. 30138293, VWR International, Fontenay-sous-Bois, Frankreich)
- Natriumcarbonat, wasserfrei, p. a., ≥ 99,9 % (z. B. Art.-Nr. 1.06392.1000, Fa. Merck KGaA, 64293 Darmstadt)
- Natriumhydrogencarbonat, p. a., > 99,5 % (z. B. Art.-Nr. 1.06329.1000, Fa. Merck KGaA, 64293 Darmstadt)
- Anionen-Multielement IC-Standard-Lösung, Fluorid (5 mg/l), Chlorid (10 mg/l), Nitrit (15 mg/l), Bromid (25 mg/l), Nitrat (25 mg/l), Phosphat (40 mg/l), Sulfat (30 mg/l) in Wasser (z. B. Roti Star Art.-Nr. 2668.2, Fa. Carl Roth GmbH + Co. KG, 76231 Karlsruhe)
- Reinstwasser (ρ ≥ 18,2 MΩ × cm bei 25 °C)

### 4.3 Lösungen

Unter Verwendung der in Abschnitt 4.2 aufgeführten Chemikalien werden folgende im Kühlschrank bei +4 ℃ über mindestens 3 Monate haltbare Lösungen hergestellt:

**Eluent-Stammlösung:** (0,62 mol Natriumcarbonat/l und 0,069 mol Natriumhydrogencarbonat/l in Wasser)

13,14 g Natriumcarbonat und 1,15 g Natriumhydrogencarbonat werden in einen 200-ml-Messkolben eingewogen, in Reinstwasser gelöst und der Messkolben bis zur Marke mit Reinstwasser aufgefüllt.

**Eluent: (**3,1 mmol Natriumcarbonat/l und 0,35 mmol Natriumhydrogencarbonat/l in Wasser)

In einen 2-l-Messkolben, in dem ca. 500 ml Reinstwasser vorgelegt wurden, werden 10 ml der Eluent-Stammlösung zugegeben. Danach wird der Messkolben mit Reinstwasser bis zur Marke aufgefüllt und geschüttelt.

### 4.4 Kalibrierstandards

Es werden zehn Kalibrierstandards durch Verdünnung der Anionen-Multielement IC-Standard-Lösung (siehe Abschnitt 4.2) hergestellt. Dazu werden mit Hilfe von Kolbenhubpipetten die in Tabelle 2 aufgeführten Volumina der Anionen-Multielement IC-Standard-Lösung in jeweils einen 10-ml-Messkolben gegeben und nachfolgend mit Reinstwasser bis zur Marke aufgefüllt. Die unverdünnte Originallösung stellt den höchsten Kalibrierstandard „XI“ dar.

**Tab.2:** Pipettierschema zur Herstellung der Kalibrierlösungen und deren Konzentrationen

Kalibrierstandard	Volumen Anionen-Multielement IC-Standard-Lösung [ml]	Endvolumen [ml]	Konzentration Nitrit [mg/l]	Konzentration Nitrat [mg/l]
I	0,050	10	0,0750	0,125
II	0,100	10	0,150	0,250
III	0,250	10	0,375	0,625
IV	0,500	10	0,750	1,25
V	0,750	10	1,13	1,88
VI	1,00	10	1,50	2,50
VII	2,00	10	3,00	5,00
VIII	4,00	10	6,00	10,0
IX	6,00	10	9,00	15,0
X	8,00	10	12,0	20,0
XI	10,0	10	15,0	25,0

Die Kalibrierstandards sind dunkel und gekühlt bei ca. 4 °C gelagert zwei Wochen verwendbar.

### 4.5 Kontrollstandards

Die Kalibrierung wird über eine Kontrollmessung mit der unverdünnten Anionen-Multielement IC-Standard-Lösung (siehe Abschnitt 4.4, vgl. Kalibrierstandard XI) abgesichert. Die Abweichung darf für Nitrit und Nitrat nicht größer als ± 5 % sein.

## 5 Probenahme und Probenaufbereitung

### 5.1 Vorbereitung der Probenträger

Die Röhrchen für die Probenahme enthalten ein mit TEA imprägniertes Al_2_O_3_-Trägermaterial. Das Trägermaterial wird vor dem Imprägnieren bei 850 °C für zwei Stunden in einem Porzellantiegel geglüht und anschließend auf Raumtemperatur abkühlen gelassen. Das geglühte Trägermaterial kann in einem verschlossenen Aufbewahrungs­gefäß aus Glas aufbewahrt und erst zu einem späteren Zeitpunkt imprägniert werden. Zur Imprägnierung werden in einem 1-l-Rotationsverdampferkolben 300 ml Reinstwasser eingefüllt und 10 ml TEA mittels Einmalspritze hinzugegeben. Anschließend werden 120 g des geglühten Al_2_O_3_-Trägermaterials hinzugegeben. Direkt nach dem Mischvorgang wird das Wasser unter langsamen Drehen mittels Rotationsverdampfer bei einer Wasserbadtemperatur von 65 °C und einem Vakuum von ca. 20 mbar bis zur Trockene entfernt. Dieser Prozess ist nach ca. zwei Stunden beendet. Das schüttfähige, imprägnierte Al_2_O_3_-Trägermaterial wird entweder zum Aufbewahren in ein Vorratsgefäß (Glas mit Verschluss) überführt oder direkt in die Probenahmeröhrchen eingefüllt. Das imprägnierte Trägermaterial ist mindestens 3 Monate lagerfähig. Eine längere Lagerdauer ist zu überprüfen.

Vor der Probenahme werden die Probenträger vorbereitet. Dazu werden die in Abschnitt 4.1 aufgeführten Materialien entsprechend der Abbildung 1 zusammengestellt. Am unteren Ende des Glasröhrchens [1] wird ein PTFE-Stopfen mit Bohrung [2], der mit zwei Viton-O-Ringen zum Fixieren versehen ist, eingesetzt. In [1] wird ein Stützsieb aus Kunststoff [4] eingesetzt, das mittels zweier Viton-O-Ringe fixiert wird. Pro Probenträger werden 4,0 bis 4,5 g des imprägnierten Al_2_O_3_-Trägermaterials mit Hilfe z. B. eines 50-ml-Becherglases und ggf. mit einem Trichter in das Glasröhrchen gefüllt. Das obere Ende wird mit den zwei Dichtungsringen und einem weiteren Stützsieb [4] verschlossen. Der Probenträger wird bis zur Probenahme mit Verschlusskappen aus PE verschlossen.

**Abb.2:**
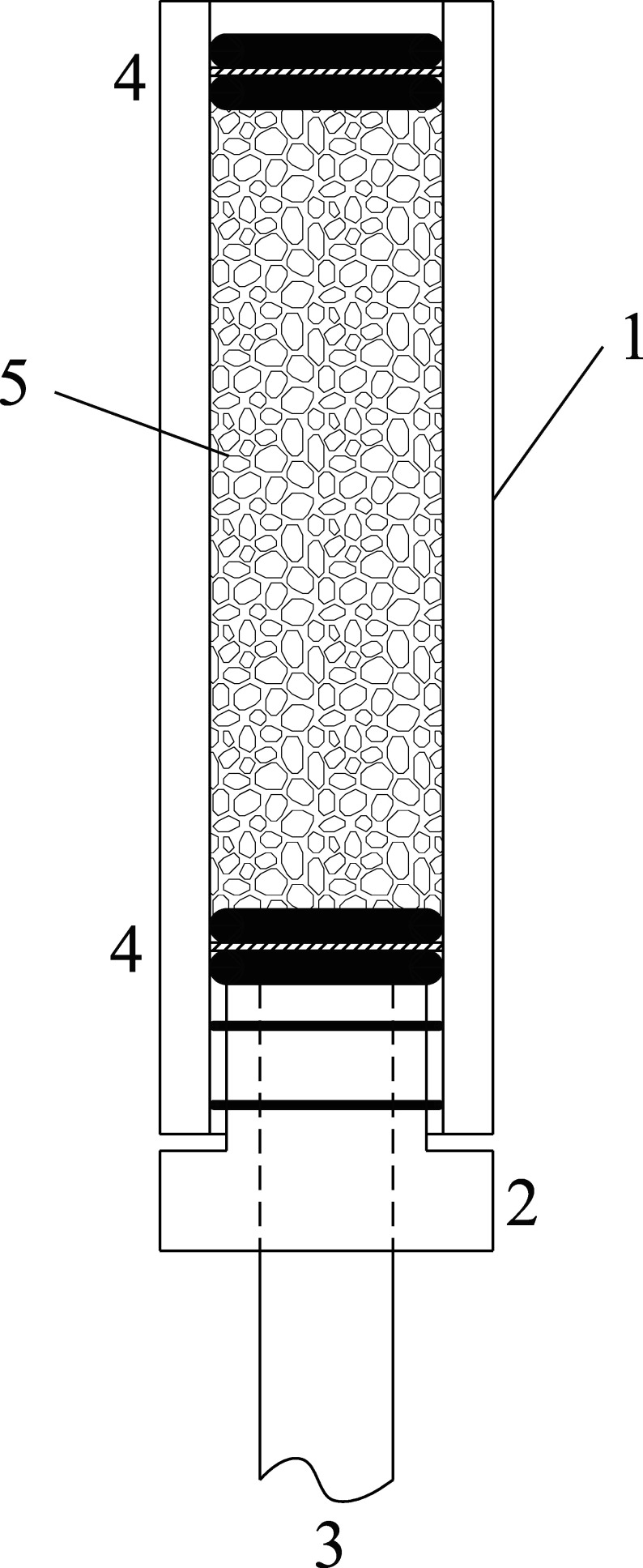
Schematische Zeichnung eines zusammengesetzten Probenträgers bestehend aus 1) Glasröhrchen, 2) PTFE-Stopfen mit zwei kleinen Viton-O-Ringen, 3) eingestecktem 8-mm-Schlauch, 4) Stützsieb aus Kunststoff zwischen zwei Viton-O-Ringen und 5) mit TEA imprägniertem Al_2_O_3_-Tägermaterial

### 5.2 Probenahme

Die Probenahme kann sowohl ortsfest als auch personengetragen erfolgen. Bei personengetragener Probenahme erfolgt diese senkrecht im Atembereich. Es ist darauf zu achten, dass die Öffnung des Probenahmeröhrchens frei zugänglich ist.

Direkt vor der Probenahme werden zunächst die Verschlusskappen vom Glasröhrchen entfernt. In die Bohrung von [2] wird ein Verbindungsschlauch mit 8 mm Außendurchmesser [3] eingeschoben. Dieser wird über einen Verbindungs­schlauch mit 8 mm Innendurchmesser mit der Probenahmepumpe verbunden. Mit Hilfe der durchflussstabilisierten Probenahmepumpe wird die Probeluft mit einem Volumenstrom von 1,8 l/min durch das Probenahmesystem gesaugt. Bei zwei Stunden Probenahme entspricht dies einem Probeluftvolumen von 216 l. Die für die Bestimmung der Luft­konzentration wichtigen Parameter (Probeluftvolumen, Temperatur, Luftdruck und relative Luftfeuchte) werden im Probenahmeprotokoll vermerkt.

Nach Beendigung der Probenahme ist der Volumenstrom auf Konstanz zu überprüfen. Ist die Abweichung vom eingestellten Volumenstrom größer als ± 5 %, wird empfohlen, die Probe zu verwerfen (DIN 2023). Die Röhrchen werden mit den Verschlusskappen dicht verschlossen und ins Labor transportiert. Die Proben sollten innerhalb von sieben Tagen aufgearbeitet werden.

Zu jeder Probenserie ist eine Blindprobe („Field Blank“) mitzuführen. Diese unterscheidet sich von der Analysenprobe lediglich darin, dass keine Probeluft durch den Filter gesaugt wurde. Die Blindprobe wird analog den Proben gelagert und aufbereitet.

### 5.3 Probenaufbereitung

Die Verschlusskappe wird auf der Anströmseite (oberes Ende) des Glasröhrchens entfernt, das Stützsieb mit einer Pinzette entnommen und das Al_2_O_3_-Trägermaterial in ein 50-ml-Kunststoffgefäß (DigiTube) überführt.

Die Al_2_O_3_-Kugeln können elektrostatisch aufgeladen sein. Ein Antistatikgerät reduziert die statische Aufladung und erleichtert die Überführung der Kugeln. Mit einem Dispenser werden 17 ml Reinstwasser hinzugegeben. Das Gefäß wird mit einem Deckel verschlossen und für ca. eine Minute manuell leicht geschüttelt, für eine Stunde stehen gelassen und dann nochmals kurz manuell geschüttelt. Das Gefäß wird zum Absetzen des suspendierten Feststoffs für eine weitere Stunde nicht bewegt.

Von der überstehenden Flüssigkeit werden ca. 4 ml mit einer 10-ml-Einmalspitze mit aufgesetzter Einmalkanüle entnommen. Die Kanüle wird entfernt und durch einen Spritzenvorsatzfilter ersetzt. Durch das Filter werden etwa 0,5 ml der Flüssigkeit filtriert und verworfen. Weitere ca. 2,5 ml werden durch dasselbe Filter in ein Autosamplergefäß filtriert. Das Autosamplergefäß wird verschlossen und im Autosampler für die IC-Analyse positioniert. Das Probegefäß mit der Restflüssigkeit wird verschlossen und als Rückstellprobe im Kühlschrank aufbewahrt.

*Hinweis: Bei der Desorption wird sehr feiner Abrieb vom Trägermaterial gelöst, der sich nur schwer aus der Lösung abtrennen lässt. Die verwendeten Spritzenvorsatzfilter verstopfen sehr schnell und es ist entsprechend viel Kraft für die Filtration nötig.*

## 6 Instrumentelle Arbeitsbedingungen

**Table:** 

**Gerät:**	Ionenchromatographiesystem (IC) mit Entgaser, Säulenofen, Autosampler, chemischer und CO_2_-Suppression
**Vorsäule:**	Metrosep A Supp 5 Guard/4.0, ID 4 mm, L 5 mm
**Trennsäule:**	Metrosep A Supp 5-250/4.0, ID 4 mm, L 250 mm
**Säulentemperatur:**	45 °C
**Detektor:**	Leitfähigkeitsdetektor
**Eluent:**	3,1 mmol Natriumcarbonat/l und 0,35 mmol Natriumhydrogencarbonat/l, isokratisch
**Flussrate:**	0,7 ml/min
**Injektionsvolumen:**	20 µl
**Laufzeit:**	42 min

Unter den angegebenen Bedingungen hat Nitrit eine Retentionszeit von ca. 11,4 und Nitrat von ca. 15,0 Minuten.

## 7 Analytische Bestimmung

Zur analytischen Bestimmung werden jeweils 20 μl der nach Abschnitt 5.3 aufbereiteten Proben in das Ionenchromato­graphiesystem injiziert und unter den in Abschnitt 6 angegebenen Bedingungen analysiert. Der Gehalt der beiden Analyten Nitrat und Nitrit wird anhand zweier getrennter Kalibrierkurven bestimmt (siehe Abschnitt 8). 

Liegen die ermittelten Konzentrationen oberhalb des Kalibrierbereiches, so sind geeignete Verdünnungen mit dem Eluent herzustellen und diese nochmals zu analysieren. Des Weiteren werden die aufbereitete Blindprobe („Field Blank“) und der Reagenzienblindwert („Lab Blank“) analog den Analysenproben analysiert.

## 8 Kalibrierung

### Externe Kalibrierung

Zur Erstellung der Kalibrierfunktionen werden die unter Abschnitt 4.4 beschriebenen Kalibrierstandards entsprechend den Abschnitten 6 und 7 analysiert. Die ermittelten Peakflächen werden gegen die jeweiligen Konzentrationen aufgetragen. Die Kalibrierfunktionen sind im untersuchten Konzentrationsbereich linear.

Zur Überprüfung der Kalibrierfunktionen wird arbeitstäglich der unter Abschnitt 4.5 beschriebene Kontrollstandard analysiert. Die Kalibrierung ist neu zu erstellen, wenn sich die analytischen Bedingungen ändern oder die Qualitäts­kontrolle dazu Anlass gibt.

## 9 Berechnung des Analysenergebnisses

Die Berechnung der Konzentration von Stickstoffdioxid in der Luft am Arbeitsplatz erfolgt über die Bestimmung der Konzentrationen von Nitrit- und Nitrationen in der Analysenlösung. Anhand der Peakflächen der Signale von Nitrat und Nitrit werden mit Hilfe der Datenauswerteinheit und der jeweilig bestimmten Kalibrierfunktion die zugehörigen Konzentrationen *c(NO*_*2*_^*–*^*)* und *c(NO*_*3*_^*–*^*) *in mg/l ermittelt. Die pro Probenträger gesammelte Menge an Stickstoffdioxid wird unter Berücksichtigung der Blindwerte für beide Ionen und des Desorptionsvolumens unter Verwendung von Gleichung 1 berechnet.


(1)

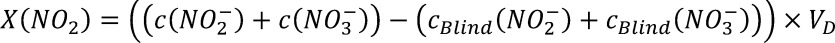



Es bedeuten: 

**Table:** 

*X(NO_2_)*	Masse von Stickstoffdioxid pro Probenträger in mg
*c(NO_2_^–^)*	Konzentration von Nitrit in der Messlösung in mg/l
*c(NO_3_^–^)*	Konzentration von Nitrat in der Messlösung in mg/l
*c_Blind_(NO_2_^–^)*	Konzentration von Nitrit des Field Blanks (Mittelwert) in mg/l
*c_Blind_(NO_3_^–^)*	Konzentration von Nitrat des Field Blanks (Mittelwert) in mg/l
*V_D_*	Volumen des Eluats in Liter (hier 0,017 l)

Unter Berücksichtigung des Probeluftvolumens und der Wiederfindung wird die Konzentration von Stickstoffdioxid in der Luft am Arbeitsplatz gemäß Gleichung 2 berechnet. Wenn eine Wiederfindung von 100±5 % im Bereich von einem Zehntel bis zum Doppelten des Grenzwertes ermittelt wurde, ist keine Korrektur in Gleichung 2 vorzunehmen.


(2)

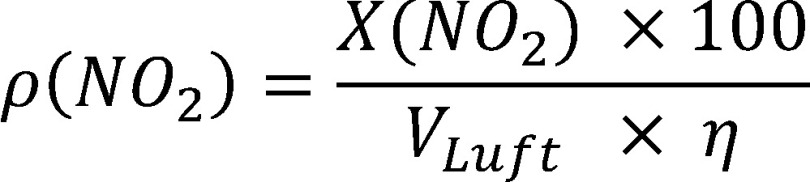



Es bedeuten: 

**Table:** 

*ρ(NO_2_)*	Massenkonzentration von Stickstoffdioxid in der Luftprobe in mg/m^3^
*V_Luft_*	Probeluftvolumen in m^3^ (ermittelt aus Volumenstrom und Probenahmedauer, hier bei 2-stündiger Probenahmedauer 0,216 m^3^)
*ƞ*	Wiederfindung in %

Zur Umrechnung auf 20 °C und 1013 hPa gilt Gleichung 3:


(3)

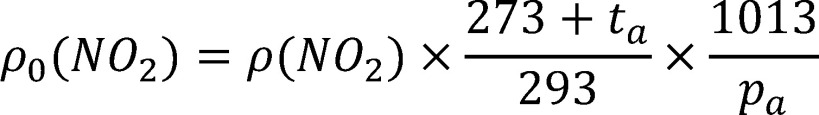



Es bedeuten: 

**Table:** 

*ρ(NO_2_)*	Massenkonzentration der Substanz in der Luftprobe in mg/m^3^ bezogen auf *t_a_* und *p_a_*
*ρ_0_(NO_2_)*	Massenkonzentration der Substanz in mg/m^3^ bezogen auf 20 °C und 1013 hPa
*t_a_*	Temperatur während der Probenahme in °C
*p_a_*	Luftdruck während der Probenahme in hPa

## 10 Beurteilung des Verfahrens

Die Kenndaten der Methode wurden gemäß DIN EN 482 (DIN 2021 a), DIN EN ISO 22065 (DIN 2021 b) und DIN 32645 (DIN 2008) ermittelt. Es wurde eine vollständige Validierung der Methode durchgeführt.

Für die Validierung des Verfahrens wurden Probenträger mit Prüfgas belegt. Dazu wurden in einem Expositionslabor Atmosphären definierter Konzentration erzeugt und Probenahmeversuche durchgeführt. Der Versuchsaufbau zur Erzeugung der Atmosphäre ist im Detail in Monsé et al. (2020) beschrieben. Die Dosierung von 0,095 mg/m^3^ (0,05 ml/m^3^), entsprechend einem Zehntel des AGW, war in diesem Labor technisch nicht durchführbar. Stattdessen wurde Prüfgas in der jeweils doppelten Konzentration (0,2 × AGW, 2 × AGW, 4 × AGW) für jeweils eine Stunde (halbe empfohlene Messzeit) mit einem Volumenstrom von 1,8 l/min durch die Probenträger gesaugt. Umgerechnet auf eine zweistündige Probenahme und ein Probeluftvolumen von 216 l entsprechen diese Belegungen Luftkonzentrationen an Stickstoffdioxid von 0,095 mg/m^3^, 0,95 mg/m^3^ und 1,9 mg/m^3^ (0,050 ml/m^3^, 0,50 ml/m^3^ und 1,0 ml/m^3^), was einem Zehntel des AGW, dem AGW und dem Doppelten des AGW entspricht. Anschließend wurden die Sammelröhrchen allen Schritten der Aufbereitung, wie unter dem Abschnitt 5.3 beschrieben, unterworfen und die Probelösungen nach der Aufbereitung gemäß der Abschnitte 6 und 7 analysiert.

### 10.1 Präzision, Wiederfindung und erweiterte Messunsicherheit

Zur Ermittlung der Präzision und der erweiterten Messunsicherheit wurden jeweils sechs Probenträger pro untersuchter Konzentration, wie unter Abschnitt 10 beschrieben ist, vorbereitet und anschließend analysiert.

Die ermittelten Präzisionsdaten, Wiederfindungen und erweiterten Messunsicherheiten sind in Tabelle 3 aufgeführt. Die Präzisions- und Wiederfindungsdaten beziehen sich auf die aus der Summe von Nitrat und Nitrit ermittelte Menge an Stickstoffdioxid.

**Tab.3:** Standardabweichung (rel.) und erweiterte Messunsicherheit *U *für n = 6 Bestimmungen

Konzentration^a)^ [mg NO_2_/m^3^] ([ml NO_2_/m^3^])	Wiederfindung [%]	Standardabweichung (rel.) [%]	Erw. Messunsicherheit *U* [%]
0,095 (0,050)	99,4	5,0	28,5
0,95 (0,50)	115,1	3,6	28,3
1,9 (1,0)	104,8	3,9	28,4

^a)^ die Konzentration ergibt sich für eine zweistündige Probenahme bei einem Volumenstrom von 1,8 l/min

Die erweiterte Messunsicherheit wurde unter Abschätzung aller relevanten Einflussgrößen ermittelt. Die Ergebnis­unsicherheit umfasst zwei wesentliche Beiträge, die Unsicherheitskomponenten der Probenahme und der Analyse. Dazu wurde die Excel-Anwendung des IFA zur Berechnung der erweiterten Messunsicherheit unter Einbeziehung eines Schätzwertes von 2 % für die Unsicherheit auf die Kalibriergeraden verwendet (IFA o.J.; DIN 2021 b).

Die Kombination aller Unsicherheitsbeiträge führt zu konzentrationsabhängigen kombinierten Messunsicherheiten des Gesamtverfahrens. Durch Multiplikation mit dem Erweiterungsfaktor k = 2 erhält man die in Tabelle 3 angege­benen Werte der erweiterten Messunsicherheit für das Gesamtverfahren.

Die berechnete erweiterte Messunsicherheit bei einer Probenahmedauer von 15 Minuten (Kurzzeitwert) beträgt maximal 29,0 %.

### 10.2 Einfluss der Luftfeuchte

Der Einfluss der Luftfeuchte wurde bei Konzentrationen von einem Zehntel und dem Doppelten des AGW bei relativen Luftfeuchten von ca. 35 % und 65 % untersucht. Dazu wurden jeweils vier Probenträger pro Konzentration und eingestellter Luftfeuchte, wie in Abschnitt 10 beschrieben, mit Prüfgas belegt. Die Probenträger wurden entsprechend den Abschnitten 5.2, 6 und 7 aufgearbeitet und analysiert. Dabei konnte kein Einfluss der relativen Luftfeuchte auf die Wiederfindung nachgewiesen werden.

### 10.3 Bestimmungsgrenze

Die Ermittlung der Bestimmungsgrenzen von Nitrit und Nitrat erfolgte aus je einer äquidistanten 10-Punkt-Kalibrierung im unteren Konzentrationsbereich gemäß DIN 32645 (DIN 2008).

Die Standards für die 10-Punkt-Kalibrierung wurden durch Verdünnung der Anionen-Multielement IC-Standard-Lösung hergestellt. Dazu wurden in 10-ml-Messkolben, in die jeweils 5 ml Eluent vorgelegt wurde, die in Tabelle 4 aufgeführten Volumina der Standard-Lösung gegeben. Anschließend wurden die Kolben mit Eluent bis zur Marke aufgefüllt und geschüttelt. Tabelle 4 enthält die Konzentrationen der zehn Kalibrierstandards.

**Tab.4:** Pipettierschema zur Herstellung der zehn Kalibrierstandards im unteren Konzentrationsbereich für die Ermittlung der Bestimmungsgrenze

Kalibrierstandard	Volumen Anionen-Multielement IC-Standard-Lösung [µl]	Endvolumen [ml]	Konzentration Nitrit [mg/l]	Konzentration Nitrat [mg/l]
I	100	10	0,150	0,250
II	200	10	0,300	0,500
III	300	10	0,451	0,750
IV	400	10	0,601	1,00
V	500	10	0,751	1,25
VI	600	10	0,901	1,50
VII	700	10	1,05	1,75
VIII	800	10	1,20	2,00
IX	900	10	1,35	2,25
X	1000	10	1,50	2,50

Bei einem Vertrauensbereich von 95 % ergibt sich eine absolute Bestimmungsgrenze von 1,9 µg NO_2_ pro Probenträger. Die entspricht für ein Probeluftvolumen von 216 Litern (1,8 l/min und 2 h Probenahme) und einem Eluentvolumen von 17 ml 0,009 mg NO_2_/m^3^ (0,005 ml/m^3^).

### 10.4 Kapazität des Probenahmesystems

Zur Bestimmung des Durchbruchverhaltens des eingesetzten Probenahmesystems wurde im Expositionslabor Prüfgas einer Konzentration von 3,8 mg/m^3^ (2,0 ml/m^3^) hergestellt und mit einem Volumenstrom von 1,8 l/min durch den Probenträger gesaugt. Zur Feststellung eines Durchbruchs wurde die durch das Probenahmesystem gesaugte Luft per Online-Massenspektrometrie im chemischen Ionisationsmodus (AirSense, Fa. MS4-Analysentechnik GmbH, 35519 Rockenberg) gemessen. Auch nach vier Stunden konnte kein Anstieg des Messsignals beobachtet werden. Somit ist das Probenahmesystem auch für eine vierstündige Probenahme beim Vierfachen des AGW bzw. MAK-Werts geeignet.

### 10.5 Lagerfähigkeit

Zur Bestimmung der Lagerfähigkeit wurde durch je acht Probenträger mit Konzentrationen von einem Zehntel und dem Doppelten des AGW, wie in Abschnitt 10 beschrieben, beaufschlagt. Vier Probenträger je Konzentration wurden direkt gemäß den Abschnitten 5, 6 und 7 aufgearbeitet und analysiert. Die Inhalte der weiteren vier Probenahmeröhrchen pro Konzentration wurden in 50-ml-Gefäße (DigiTube) überführt, mit 17 ml Reinstwasser versetzt, luftdicht verschlossen und für 4 Wochen lang verschlossen im Kühlschrank bei ca. 4 °C gelagert. Anschließend wurden die Extrakte gemäß den Abschnitten 5, 6 und 7 aufgearbeitet und analysiert.

Die mittlere Wiederfindung nach vier Wochen Lagerung im Kühlschrank betrug 92,0 %. Die verminderte Wiederfindung bei längerer Lagerung muss somit in der Berechnung des Analysenergebnisses berücksichtigt werden.

### 10.6 Selektivität und Querempfindlichkeiten

**Abb.3:**
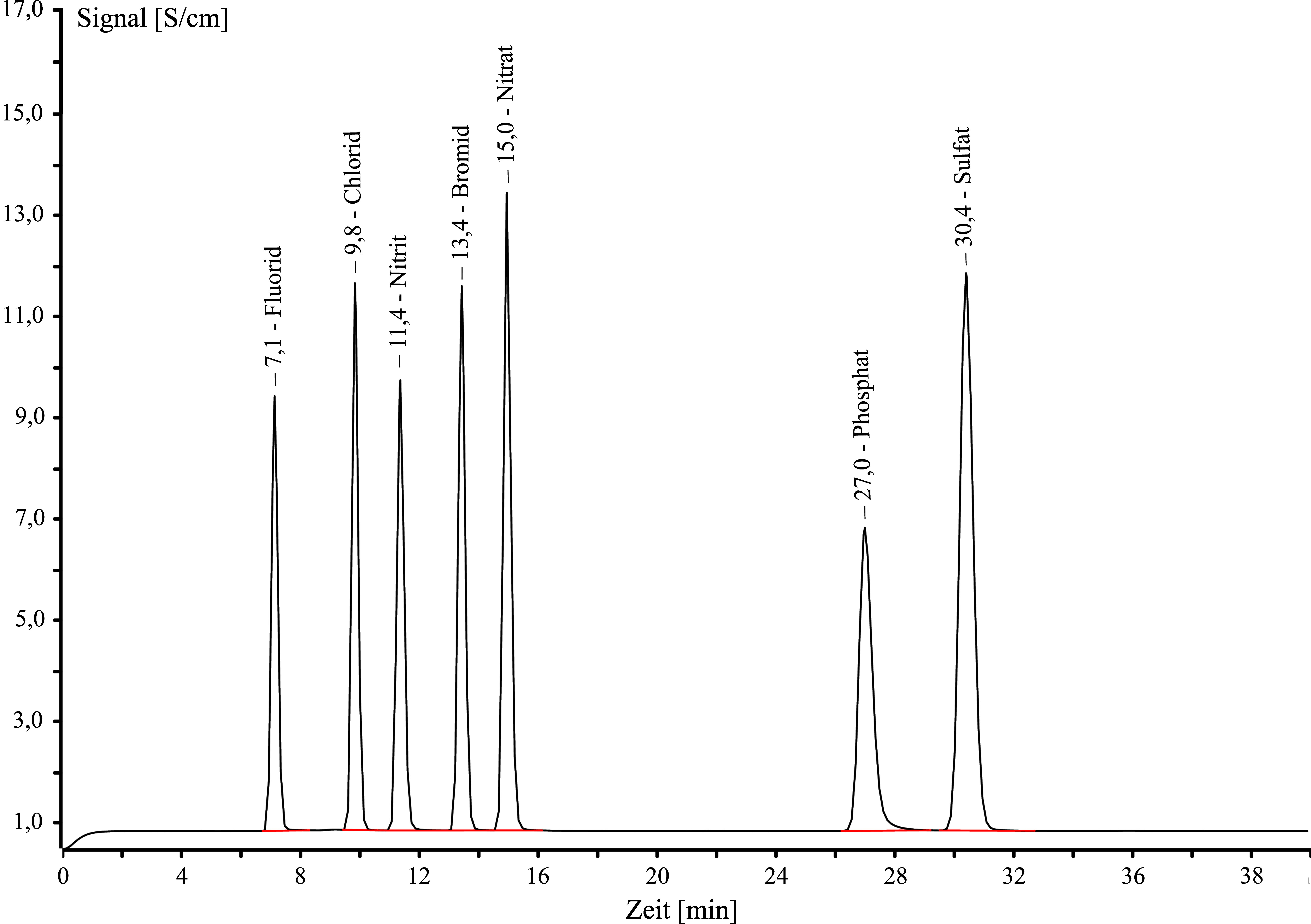
Beispielchromatogramm für die ionenchromatographische Trennung von Nitrit und Nitrat von den weiteren häufig vorkommenden Anionen Fluorid, Chlorid, Bromid, Phosphat und Sulfat

Das Analysenverfahren mittels IC ist unter den angegebenen Arbeitsbedingungen spezifisch und robust. Es konnten keine Störungen beobachtet werden. Eine chromatographische Unterscheidung zwischen Nitrit, Nitrat und weitere Anionen ist gewährleistet (siehe Abbildung 3).

Neben der gewünschten Reaktion zwischen Stickstoffdioxid und TEA können auch andere Moleküle mit TEA reagieren und Nitrit- und Nitrationen bilden bzw. abscheiden. Die wichtigsten potenziellen positiven Interferenzen sind, abgesehen von der Reaktion von NO mit Ozon (O_3_) während der Probenahme, Peroxyacetylnitrat (PAN) [2278-22-0], salpetrige Säure (HNO_2_) [7782-77-6] (Cape 2009) und Salpetersäure (HNO_3_) [7697-37-2]. 

Partikulär auftretende Nitrite und Nitrate können bei der Probenahme ebenfalls miterfasst werden und führen somit zu falsch positiven Messergebnissen. Es wird empfohlen, sofern partikulär vorkommende Salze der salpetrigen Säure und der Salpetersäure nicht ausgeschlossen werden können, ein geeignetes Filter (z. B. PTFE) in einem Filterhalter möglichst totvolumenarm mittels eines Silikonschlauches mit dem Sammelröhrchen zu koppeln. Das vorgeschaltete Filter wird bei der Analytik nicht berücksichtigt. Der zusätzliche Druckverlust ist bei der Einstellung der Probenahmepumpe zu berücksichtigen.

## 11 Diskussion

Das beschriebene Messverfahren ermöglicht die Bestimmung von Stickstoffdioxid in der Luft am Arbeitsplatz in einem Konzentrationsbereich von einem Zehntel bis zum Doppelten derzeit gültigen AGW bzw. MAK-Wertes von 0,95 mg/m^3^ (0,5 ml/m^3^). Das Messverfahren ist geeignet, um die Einhaltung des Kurzzeitwertes zu überprüfen.
